# Towards development and validation of an intraoperative assessment tool for robot-assisted radical prostatectomy training: results of a Delphi study

**DOI:** 10.1590/S1677-5538.IBJU.2016.0420

**Published:** 2017

**Authors:** Christopher Morris, Jen Hoogenes, Bobby Shayegan, Edward D. Matsumoto

**Affiliations:** 1 Department of Surgery, Division of Urology, McMaster University, Hamilton, ON, Canada

**Keywords:** Delphi Technique, Prostatectomy, Robotic Surgical Procedures

## Abstract

**Introduction:**

As urology training shifts toward competency-based frameworks, the need for tools for high stakes assessment of trainees is crucial. Validated assessment metrics are lacking for many robot-assisted radical prostatectomy (RARP). As it is quickly becoming the gold standard for treatment of localized prostate cancer, the development and validation of a RARP assessment tool for training is timely.

**Materials and methods:**

We recruited 13 expert RARP surgeons from the United States and Canada to serve as our Delphi panel. Using an initial inventory developed via a modified Delphi process with urology residents, fellows, and staff at our institution, panelists iteratively rated each step and sub-step on a 5-point Likert scale of agreement for inclusion in the final assessment tool. Qualitative feedback was elicited for each item to determine proper step placement, wording, and suggestions.

**Results:**

Panelist’s responses were compiled and the inventory was edited through three iterations, after which 100% consensus was achieved. The initial inventory steps were decreased by 13% and a skip pattern was incorporated. The final RARP stepwise inventory was comprised of 13 critical steps with 52 sub-steps. There was no attrition throughout the Delphi process.

**Conclusions:**

Our Delphi study resulted in a comprehensive inventory of intraoperative RARP steps with excellent consensus. This final inventory will be used to develop a valid and psychometrically sound intraoperative assessment tool for use during RARP training and evaluation, with the aim of increasing competency of all trainees.

## INTRODUCTION

Surgical education has recently undergone a paradigm shift towards competency-based frameworks for surgical training and evaluation. A need for improved training, certification, and recertification in Urology has been recognized. As such, health care regulatory bodies in the United States, Canada, and Europe are revising curricula with a new focus on what trainees should know in order to be deemed competent (1-3). With this shift, the need for valid, reliable, and feasible assessment tools exists; however, there is a paucity in many surgical specialities. Robot-assisted urologic surgery (RUS) is rapidly gaining in accessibility and popularity, with robot-assisted radical prostatectomy (RARP) now considered the frontline treatment for clinically localized prostate cancer. Yet, no standardized training or evaluation models have been developed for RARP. While its anatomic technique has been well- described by Menon and colleagues since its initiation in 2000, with their most recent report in 2012 (4), reliable objective metrics for trainee evaluation of RARP have not been established.

The daVinci Surgical System® (Intuitive Surgical, Sunnyvale, CA) boasts multiple technical advantages over traditional laparoscopic and open radical prostatectomy, and affords a more practicable and ergonomic environment to enhance the learning curve (5, 6). Evidence suggests that RARP produces more favorable patient outcomes when compared to its traditional counterparts (7, 8). As RARP is the most commonly performed robotic procedure worldwide (9), and RUSs such as radical and partial nephrectomy and pyeloplasty are becoming more prevalent, proposed best practices, training and credentialing criteria, standard operating practices (SOPs), and frameworks for effective incorporation of robotic surgical programs into institutions is timely. The American Urological Association (AUA) has recently proposed SOPs for robotic surgery that include minimum requirements for granting urologic robotic privileges (5); however, consensus has not yet been reached for a standardized curriculum and credentialing system. Several academic centers have published their own guidelines for RUS credentialing; but again, a lack of universal consensus exists (10). Regarding RARP credentialing, Zorn and colleagues recently published recommendations on behalf of the Society of Urologic Robotic Surgery (SURS) (11), and McDougall and colleagues have established a successful mini-residency for RARP (12) that provides a framework for postgraduate teaching. More recently, best practices for RARP have been proposed (13); despite the efforts of numerous organizations, a consensus for training, credentialing, and assessment of competency for RUS, including RARP, have not yet been achieved (5).

Inanimate and virtual reality (VR) simulation has played a significant role in the training of robotic surgery. Clear benefits of simulation have been established in the literature, suggesting shorter operative time and fewer medical errors when skills transfer to the high-stakes environment of the operating room (OR) (14, 15). Further, the validity of the da Vinci Skills Simulator (Mimic™ VR software) has recently been established (16-18), providing support for an effective training platform, especially when surgical training time with the da Vinci is extremely limited. Despite preparing trainees for the robotic environment, it has not yet been unequivocally demonstrated that these simulated robotic skills can indeed transfer to the OR. Further, procedure-specific VR programs for RARP are not yet widely available.

Based on a clear need for a standardized intraoperative assessment tool for RARP to measure and establish competency during training, we set out to design a step-wise clinical assessment tool for the RARP procedure. We report on a study to address the first stage in this process, which comprises the development of an inventory of procedural RARP steps and sub-steps as defined by a panel of expert RARP surgeons via a modified Delphi process.

## MATERIALS AND METHODS

### Study Design and Population

A modified Delphi process was employed to achieve consensus of the items that expert RARP surgeons believe ought to comprise the assessment tool. The Delphi technique is an iterative structured group communication method to solicit expert opinion about new or complex problems, conducted through a series of questionnaires (typically three to four rounds) with controlled feedback each round (19). The Delphi process goal is to achieve expert consensus using qualitative and quantitative methodology. The feedback process allows and encourages participants to reassess their initial judgements and revise them throughout the iterations (20). Anonymity and confidentiality are maintained for each panel member. The controlled feedback process eliminates biases that often occur during group consensus approaches like panel meetings and focus groups. Further, Delphi statistical analysis ensures that each member’s opinions are well represented in the final iteration, as it allows for objective and impartial analysis when summarizing the data (20). We first employed a modified Delphi study with urology staff surgeons, fellows, and residents at our institution to develop a preliminary inventory of RARP steps. Following the internal process, we recruited a panel of expert RARP surgeons external to our institution to participate in a Delphi study to evaluate and edit the initial inventory, with the goal of developing a final inventory of steps and sub-steps for RARP.

### Internal Delphi Process

Following ethics approval, we recruited seven participants at our institution to serve as a Delphi panel. This included two RARP experts (performed >300 RARP cases), one urology fellow, and four senior urology residents. The fellow and residents all had extensive experience as a RARP bedside assistant and the majority had some intraoperative da Vinci console experience. Our rationale for employing the modified internal Delphi technique was to create a RARP procedural inventory from scratch, in accordance with Delphi methodology. We provided each member of the group with a RARP video from our case database and asked members to create a list of the critical steps and sub-steps of the entire procedure, referring to the video as necessary. This began by using an open-ended format followed by a checklist system for inclusion criteria. Qualitative comments were encouraged and modifications were made. Four iterations were conducted until 100% consensus was reached. It was then circulated to all members for final approval.

### External Delphi Process

The expert panel was recruited via email by two expert RARP surgeons at our institution. Twenty-nine expert RARP surgeons from Canada (17) and the United States (12) were asked to participate. Potential participants were provided with a comprehensive background of the study process, and those who chose to participate provided informed consent via by email, with the final panel totaling thirteen participants. The literature recommends that a total of ten to eighteen panel members is sufficient for consensus if the sample is homogenous (11, 21). An advanced version of the web-based SurveyMonkey® software (Palo Alto, CA) was used to create and submit each of the survey iterations. This email-based system provided us with controlled, quantitative and qualitative feedback, and allowed for analysis of data through Excel, SPSS, graphical formats, and data summaries.

Four iterations were conducted. The first round’s survey was derived from the internal Delphi’s inventory of steps. A 5-point Likert scale with a neutral option was used for each response option and panel members were instructed to rank the importance, in terms of agreement, of whether each primary step and sub-step ought to be included in a RARP assessment tool. They were also encouraged to provide feedback for each item, and overall comments at the end of the survey. Panel members had the option to anonymously contact the study team with questions during the process, and for reference, all members were provided a link to the same RARP video distributed during the internal Delphi study. The initial survey was piloted with two urologic surgeons at our institution. Following the first survey distribution, three reminders were sent at predetermined weekly intervals until all responses were received. Results of the first round were analyzed and edits to the inventory items were made based on feedback, while some sub-steps were moved in the sequence and/or eliminated based on score consensus. Each item’s consensus was based on Ulschak’s (22) criteria, whereby 80 percent of subject’s votes fall within two categories on a 5-point scale. If items fell beneath a mean of 3.0, they were either deleted from the inventory or modified as suggested by panelists. Even when consensus was reached to keep the items, several required modifications based on feedback. The final iteration’s methodology was modified per Delphi protocol to adjust to a 4-point Likert scale, eliminating the neutral response option to minimize satisficing. For consensus to be achieved on a 4-point scale, it is recommended that at least 70 percent of Delphi subjects need to rate a mean of 3.25 or higher on each item (20). Descriptive statistics were analyzed in SPSS v22® for each round of the Delphi process.

## RESULTS

Detailed demographics of the expert Delphi panel participants are described in Table-1. All thirteen participants were male and the majority were fellowship trained in robotic surgery. At the time of the study, each panelist was performing RARPs at high volume academic tertiary care centers within the U.S. or Canada.

**Table 1:** Demographics of External Delphi Panel.

	N (%)
**Sex**	
Male	13 (100)
**Age**	
35-44	10 (77)
45-54	3 (23)
**Practice Setting**	
Tertiary Care	13 (100)
Community	0 (0)
**Practice Location**	
Canada	9 (69)
USA	4 (31)
**Years in Practice**	
1-5	3 (23)
6-10	5 (38.5)
10-15	4 (30.8)
>15	1 (7.7)
**Fellowship**	
Yes	11 (84.6)
No	2 (15.4)
**Years Performing RARP**	
1-2	4 (30.8)
3-4	4 (30.8)
5-7	4 (30.8)
10	1 (7.6)
**RARP per month**	
1-5	10 (76.9)
6-10	2 (15.4)
10-15	1 (7.7)
>15	0 (0)

Results of the internal Delphi process included an overall reduction of inventory steps and sub-steps of 18% over a total of three iterations, with a fourth iteration serving to determine final agreement. The final inventory received 100% consensus and consisted of 13 critical steps and 60 sub-steps in total.

**Figure 1:**
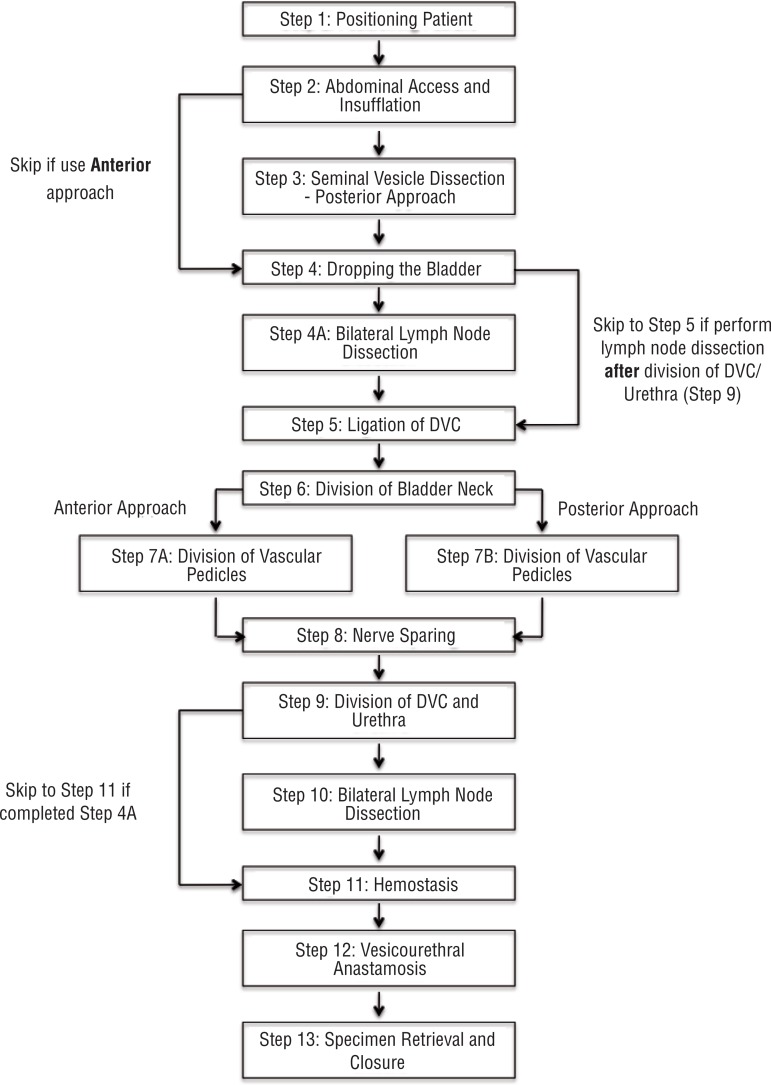
Critical steps for RARP algorithm.

**Figure 2:**
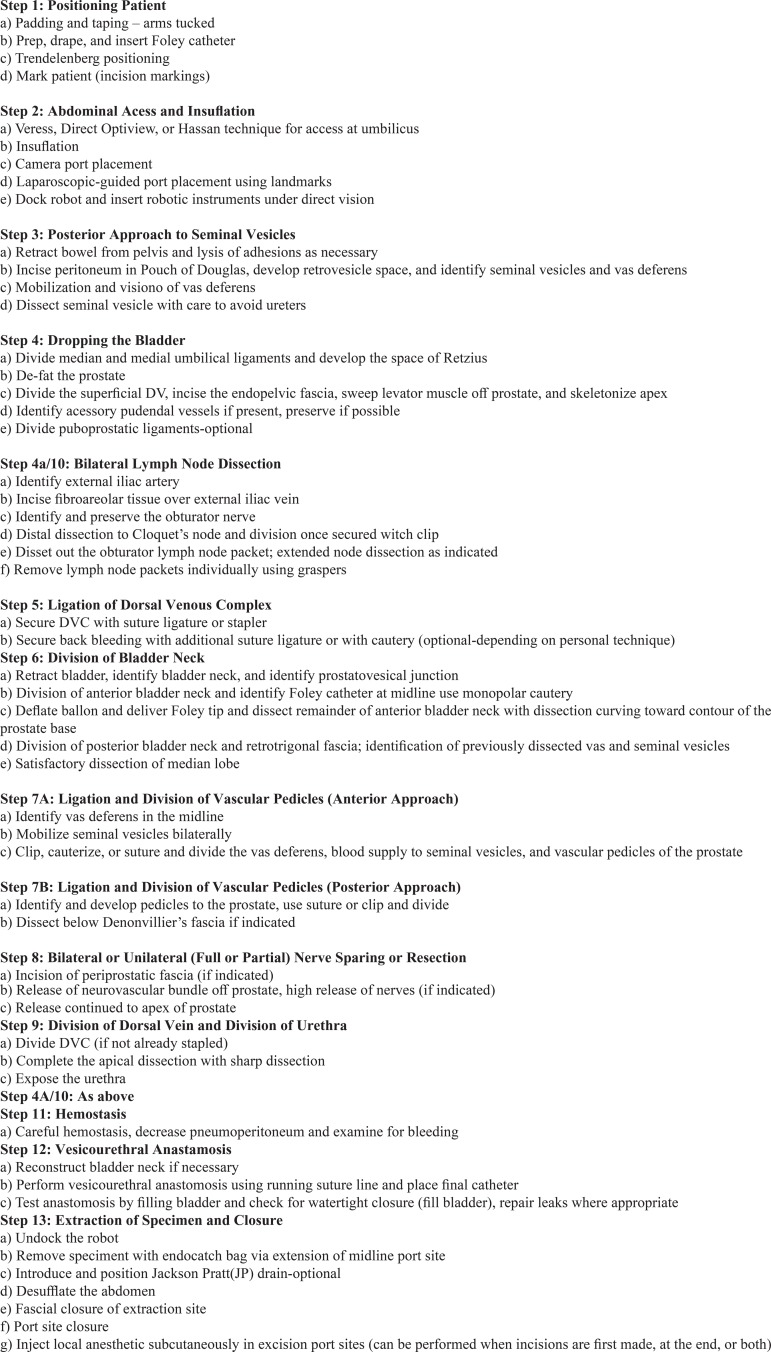

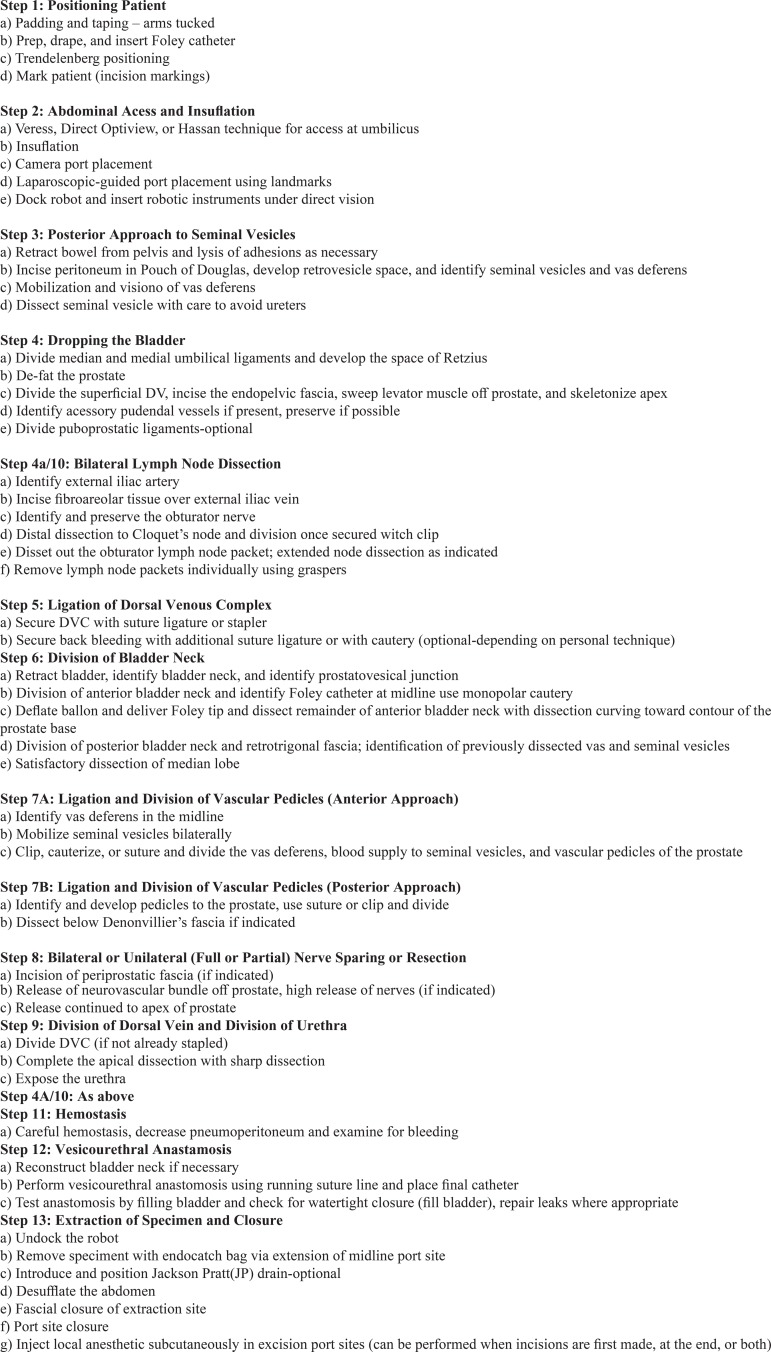
Final inventory of steps and sub-steps.

**Figure 3:**
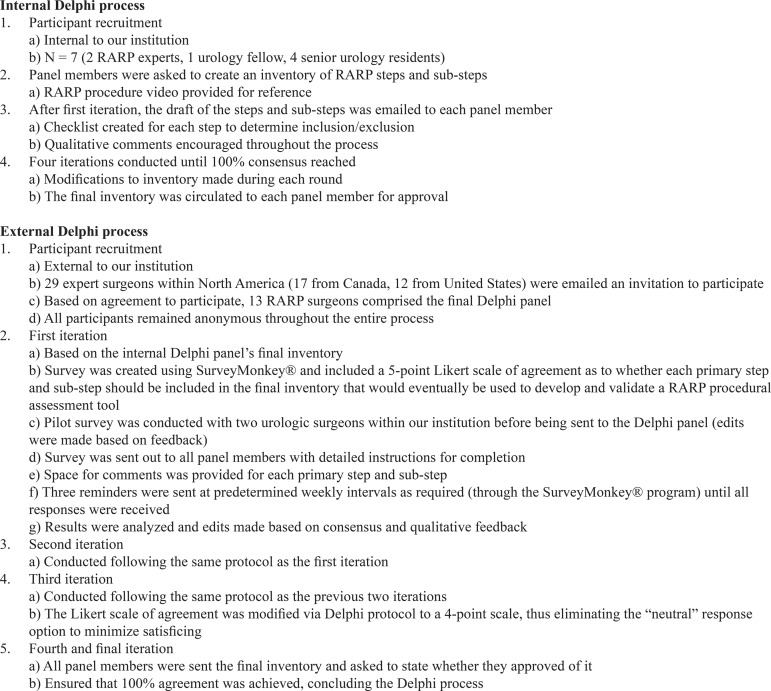
Outline of the Delphi process.

This initial inventory was used as the framework for the external Delphi study. After the first round, there was a 58% consensus of the RARP steps and sub-steps. Qualitative and quantitative feedback led to the removal of 4 sub-steps and required the addition of 2 skip patterns in the algorithm, specifically with regard to the approach to the prostate (anterior versus posterior) and timing of the lymph node dissection (Figure-1). The second iteration reached 75% consensus and further reduced the number of sub-steps to a total of 52. The third round reached 100% consensus. The fourth round served to determine whether the inventory was indeed the final version based on agreement by panel members (Figure-2). The internal and external Delphi processes are outlined in Figure-3.

## DISCUSSION

Despite the widespread use of the da Vinci Surgical System, relatively little attention has been paid to robotic curricula and assessment metrics for training (5, 10, 14, 15). Currently, there are no standardized guidelines for teaching, evaluating, and credentialing robotic surgery. In 2014, Smith and colleagues (23) reported on several consensus conferences that took place with fourteen societies in an effort to develop a standardized process for certifying the skills of robotic surgeons. This has been termed Fundamentals of Robotic Surgery (FRS), which was modeled after the Society of American Gastrointestinal and Endoscopic Surgeon’s (SAGES) validated and widely used Fundamentals of Laparoscopic Surgery (FLS) curriculum (23). However, this is generically designed for all types of robotic surgery, and therefore cannot be applied to specific procedures without first tailoring it to the procedure.

The current surgical education landscape favors global rating scales (GRSs) over checklists, as GRSs have been shown to improve validity and reliability. The recently published and validated Global Evaluative Assessment for Robotic Skills (GEARS) assessment tool (24), which was derived from the validated Global Operative Assessment of Laparoscopic Skills (GOALS) tool for laparoscopic surgery (25), expanded on the innovative work of Martin et al. to create a generalized assessment tool for robotic surgery (26). However, GEARS is not task-specific; therefore when used, it must be tailored to the task being evaluated. An example of a task-specific GRS is the recently proposed Robotic Anastomosis Competency Evaluation (RACE) that purports to assess the technical skills of performing a urethrovesical anastomosis during RARP (27). Metrics for assessing the competency of the entire RARP procedure are lacking; thus, our development of a consensus-based inventory of RARP steps is the first attempt at creating a valid and reliable stepwise RARP assessment tool for use in training. Research by Ali et al. and Schreuder et al. (28, 29) and Rashid et al. (30) have demonstrated that a proficiency-based stepwise approach to learning robotic training is both feasible and safe. Thus, instead of relying on a single GRS, our proposed assessment tool will allow surgical educators to rate trainees on each step of the RARP procedure as they progress through the learning curve.

The use of a modified Delphi methodology via survey software was ideal for gaining expert consensus on the critical main steps and the sub-steps of the RARP procedure, as it offered a systematic process for data collection. Firm timelines were used and regularly scheduled reminders were sent out during each round. We minimized attrition by selecting participants with a high interest in RARP training and by informing participants of the processes and goals of the study at the outset and by maintaining regular two-way communication. Anonymity was also preserved, allowing participants to overcome any communication barriers inherent in face-to-face interaction and focus groups, and participants were able to modify their views without the element of social pressure. Furthermore, the technique allowed for time flexibility, as respondents were able to complete their surveys on their own time. The process also afforded the respondents controlled feedback whereby they were able to see the inventory develop with each iteration, allowing them to observe that their input was leading to tangible results.

Limitations of the process included selection bias, as respondents were known to the two recruiting surgeons; however, maintaining anonymity throughout the process helped to control for this bias. Still, potential respondents may have felt social pressure to participate. Furthermore, inherent to all Delphi studies, the judgments were those of a select group of people and may not necessarily have been representative of all RARP surgeons. Additionally, Delphi methodology inherently limits or excludes outliers on a scale of an item and forces a more middle of the road consensus. This was mediated by including space for qualitative feedback for each item evaluated.

We have begun to develop an assessment tool based on the stepwise inventory, with evaluation metrics built in for each step. We have maintained a prospective database of RARP cases that will be used for rating each step. Experts will be recruited and asked to rate the endoscopic videos of resident (PGY3-5) and expert cases using the GEARS tool to assess each step. Experts will be blinded to level of training. To minimize time burden, participants will be asked to evaluate only two steps at a time until each step has been assessed. Access to the videos will be provided by a secure link sent via email. Our goal is to develop and validate a reliable stepwise RARP assessment tool based on our inventory of steps acquired during this Delphi process. This assessment tool may eventually be incorporated into residency and/or fellowship curricula for use during intraoperative RARP training. The potential for changes to the RARP inventory is indeed possible as we receive additional feedback on the evaluation tool, especially with regard to alternate means of techniques during the steps, with the potential to include issues specific to plausible patient outcomes.

## CONCLUSIONS

Our team has successfully developed an inventory of crucial steps and sub-steps for RARP based on expert consensus using Delphi methodology. We aim to develop and validate a reliable assessment tool that will be based on this stepwise inventory and can be used during intraoperative RARP training to improve competency of trainees as they learn RARP.

## ABBREVIATIONS

RARP: robot-assisted radical prostatectomy
RUS: robotic urologic surgery
SOP: standard operating practices
AUA: American Urological Association
SURS: Society of Urologic Robotic Surgery
VR: virtual reality
OR: operating room
FRS: Fundamentals of Robotic Surgery
SAGES: Society of American Gastrointestinal and Endoscopic Surgeons
FLS: Fundamentals of laparoscopic Surgery
GEARS: Global Evaluative Assessment for Robotic Skills
GRS: Global Rating Scale
GOALS: Global Operative Assessment of Laparoscopic Skills
RACE: Robotic Anastomosis Competency Evaluation

## References

[1] Ahmed K, Jawad M, Dasgupta P, Darzi A, Athanasiou T, Khan MS (2010). Assessment and maintenance of competence in urology. Nat Rev Urol.

[2] Abrams P, Brausi M, Buntrock S, Ebert T, Hashim H, Tiselius HG (2012). The future of urology. Eur Urol.

[3] Srinivasan M, Li ST, Meyers FJ, Pratt DD, Collins JB, Braddock C (2011). “Teaching as a Competency”: competencies for medical educators. Acad Med.

[4] Ghani KR, Trinh QD, Menon M (2012). Vattikuti Institute Prostatectomy-Technique in 2012. J Endourol.

[5] Ghani KR, Trinh QD, Sammon J, Jeong W, Dabaja A, Menon M (2012). Robot-assisted urological surgery: Current status and future perspectives. Arab J Urol.

[6] Palmer KJ, Lowe GJ, Coughlin GD, Patil N, Patel VR (2008). Launching a successful robotic surgery program. J Endourol.

[7] Novara G, Ficarra V, Rosen RC, Artibani W, Costello A, Eastham JA (2012). Systematic review and meta-analysis of perioperative outcomes and complications after robot-assisted radical prostatectomy. Eur Urol.

[8] Ficarra V, Cavalleri S, Novara G, Aragona M, Artibani W (2007). Evidence from robot-assisted laparoscopic radical prostatectomy: a systematic review. Eur Urol.

[9] Rocco B, Matei DV, Melegari S, Ospina JC, Mazzoleni F, Errico G (2009). Robotic vs open prostatectomy in a laparoscopically naive centre: a matched-pair analysis. BJU Int.

[10] Lee JY, Mucksavage P, Sundaram CP, McDougall EM (2011). Best practices for robotic surgery training and credentialing. J Urol.

[11] Zorn KC, Gautam G, Shalhav AL, Clayman RV, Ahlering TE, Albala DM (2009). Training, credentialing, proctoring and medicolegal risks of robotic urological surgery: recommendations of the society of urologic robotic surgeons. J Urol.

[12] McDougall EM, Corica FA, Chou DS, Abdelshehid CS, Uribe CA, Stoliar G (2006). Short-term impact of a robot-assisted laparoscopic prostatectomy ‘mini-residency’ experience on postgraduate urologists’ practice patterns. Int J Med Robot.

[13] Montorsi F, Wilson TG, Rosen RC, Ahlering TE, Artibani W, Carroll PR (2012). Best practices in robot-assisted radical prostatectomy: recommendations of the Pasadena Consensus Panel. Eur Urol.

[14] Dulan G, Rege RV, Hogg DC, Gilberg-Fisher KM, Arain NA, Tesfay ST (2012). Developing a comprehensive, proficiency-based training program for robotic surgery. Surgery.

[15] Brinkman WM, Luursema JM, Kengen B, Schout BM, Witjes JA, Bekkers RL (2013). da Vinci skills simulator for assessing learning curve and criterion-based training of robotic basic skills. Urology.

[16] Hung AJ, Patil MB, Zehnder P, Cai J, Ng CK, Aron M (2012). Concurrent and predictive validation of a novel robotic surgery simulator: a prospective, randomized study. J Urol.

[17] Hung AJ, Zehnder P, Patil MB, Cai J, Ng CK, Aron M (2011). Face, content and construct validity of a novel robotic surgery simulator. J Urol.

[18] Kenney PA, Wszolek MF, Gould JJ, Libertino JA, Moinzadeh A (2009). Face, content, and construct validity of dV-trainer, a novel virtual reality simulator for robotic surgery. Urology.

[19] Day J, Bobeva M (2005). A generic toolkitfor the successful management of Delphi studies. Electronic Journal of Business Research Methods.

[20] Hsu C, Sandford BA (2007). the Delphi technique: making sense of consensus. Practical Assessment, Research, and Evaluation.

[21] Delbecq AL, Van de Ven AH, FGustafson DH (1975). Group Techniques for Program Planning.

[22] Ulschak FL (1983). Human resource development: The theory and practice of need assessment.

[23] Smith R, Patel V, Satava R (2014). Fundamentals of robotic surgery: a course of basic robotic surgery skills based upon a 14-society consensus template of outcomes measures and curriculum development. Int J Med Robot.

[24] Goh AC, Goldfarb DW, Sander JC, Miles BJ, Dunkin BJ (2012). Global evaluative assessment of robotic skills: validation of a clinical assessment tool to measure robotic surgical skills. J Urol.

[25] Vassiliou MC, Feldman LS, Andrew CG, Bergman S, Leffondré K, Stanbridge D (2005). A global assessment tool for evaluation of intraoperative laparoscopic skills. Am J Surg.

[26] Martin JA, Regehr G, Reznick R, MacRae H, Murnaghan J, Hutchison C (1997). Objective structured assessment of technical skill (OSATS) for surgical residents. Br J Surg.

[27] Raza SJ, Field E, Jay C, Eun D, Fumo M, Hu JC (2015). Surgical competency for urethrovesical anastomosis during robot-assisted radical prostatectomy: development and validation of the robotic anastomosis competency evaluation. Urology.

[28] Ali MR, Rasmussen J, BhaskerRao B (2007). Teaching robotic surgery: a stepwise approach. Surg Endosc.

[29] Schreuder HW, Wolswijk R, Zweemer RP, Schijven MP, Verheijen RH (2012). Training and learning robotic surgery, time for a more structured approach: a systematic review. BJOG.

[30] Rashid HH, Leung YY, Rashid MJ, Oleyourryk G, Valvo JR, Eichel L (2006). Robotic surgical education: a systematic approach to training urology residents to perform robotic-assisted laparoscopic radical prostatectomy. Urology.

